# GENDERED TRAJECTORIES OF DEPRESSIVE SYMPTOMS IN OLDER KOREAN PARENTS BEREAVED OF AN ADULT CHILD

**DOI:** 10.1093/geroni/igad104.2439

**Published:** 2023-12-21

**Authors:** Sujeong Park, Jinho Kim

**Affiliations:** Korea University, Seoul, Republic of Korea; Korea University, Seoul, Republic of Korea

## Abstract

Despite the existing body of research on the impact of child bereavement, little is known about whether time to the death of an adult child is associated with changes in depressive symptoms among older Korean parents. This study examines (a) trajectories of depressive symptoms before and after the loss of an adult child and (b) whether these trajectories differ across parent-child pairs (father-son, father-daughter, mother-son, and mother-daughter). Using eight waves of the Korean Longitudinal Study of Ageing (KLoSA), the study employs fixed effects models to mitigate potential bias due to unobserved individual-level heterogeneity. The result of this study revealed that depressive symptoms increased within the first year following the loss of an adult child among bereaved parents. Considering the gender of both the parent and the deceased child, differences in psychological adjustment to bereavement were observed in different dyads. Depressive symptoms surged within the first year and persisted even beyond the fourth year of loss among daughter-bereaved fathers, while only an immediate rise in depressive symptoms within the first year of loss occurred for other pairs. Given prevailing gender role socialization in the Korean context, men’s conformity to masculinity norms and the traditional expectation for daughters to provide emotional support may contribute to the long-term increase in depressive symptoms for fathers who have lost a daughter. Thus, in societies where Confucian gender culture is ingrained as a social norm, policies for the psychological support of older parents who have lost adult children must consider the different trajectories of parent-child relationships.

